# Incidence and risk factors of inguinal hernia occurred after radical prostatectomy-comparisons of different approaches

**DOI:** 10.1186/s12893-020-00883-9

**Published:** 2020-10-02

**Authors:** Lijia Liu, Haoxiang Xu, Feng Qi, Shangqian Wang, Kamleshsingh Shadhu, Dadhija Ramlagun-Mungur, Shui Wang

**Affiliations:** 1 Department of General Surgery, The First Affiliated Hospital of Nanjing Medical University, Nanjing, Jiangsu 210029 People’s Republic of China; 2 Department of Urology, The First Affiliated Hospital of Nanjing Medical University, Nanjing, Jiangsu 210029 People’s Republic of China; 3 Medical Council of Mauritius, One Way Floreal Road, Cite Magalkhan, Floreal, Vacoas-Phoenix, Mauritius

**Keywords:** Postoperative inguinal hernia, Prostatectomy, Older age, Prostate cancer

## Abstract

**Background:**

To observe cumulative morbidity of postoperative inguinal hernia (PIH) and identify risk factors associated with its development in patients who underwent retropubic radical prostatectomy (RRP), laparoscopic prostatectomy (LRP) or robotic assisted laparoscopic prostatectomy (RALP) operation.

**Methods:**

From June 2009 to September 2016, 756 patients diagnosed with localized prostate cancer who had undergone RRP, LRP or RALP in our center were included in this study. Patients with PIH were retrospectively investigated in such factors as age, BMI, previous abdominal operations, diabetes mellitus history, hypertension history, prostate volume, previous hernia, operative methods, operative approach, preoperative Gleason, clinical T-stage, PLND situation, operative time, and estimated blood loss. Univariate and multivariate cox hazard regressions analysis were utilized to identify risk factors predisposing to PIH.

**Results:**

A total of 53 of 751(7.1%) patients developed PIH at a median follow-up period of 43 months. PIH rate in RRP was significantly higher compared to LRP and RALP group (RRP: 15.3%, LRP: 6.7%, RALP:1.9%, *P* = 0.038). Right side (69.8%) and indirect (88.8%) PIH were dominant type in hernia group. Univariate and multivariate cox hazard regressions analysis indicated that age and RRP approach were identified to be implicated to PIH [adjusted hazard ratio7.39(1.18–46.39), 2.93(95% CI 1.47–5.84)].

**Conclusions:**

RRP technique and older age, especially patients over 80 years, are associated with higher incidence for PIH development. Appropriate prophylaxis during the operation should be evaluated for those in high-risk.

## Background

The incidence of prostate cancer (PCa) has been increasing rapidly during the last decade due to widely applied prostate-specific antigen (PSA) screening [1]. To date, prostate cancer has become the most diagnosed solid malignancies among American men. In 2018, there were 164,690 estimated new cases, and 1 in every 5 newly diagnosed cancer was PCa [1], especially the localized and locally advanced PCa. Radical prostatectomy is one of the gold standard treatment for early-stage PCa, which could be operated by retropubic radical prostatectomy (RRP), laparoscopic prostatectomy (LRP) and robotic assisted laparoscopic prostatectomy (RALP) approaches. Some relatively well-studied complications such as erectile dysfunction and urinary incontinence have acquired more concerns among surgeons [2]. However, in a retrospective study,radical retropubic prostatectomy was recognized to be implicated in the occurrence of PIH [3],which may cause pain, intestinal dysfunction and even require emergency surgery owing to possible strangulated small intestine. In 1996, Regan et al. reported a 12% incidence of inguinal hernia in 6 months after RRP compared to 5% in general male population for the first time [4]. Since then,several papers had studied the cumulative PIH rates ranged from 1.5 to 50% regarding different prostatectomy methods [5–7].

The choice of surgical technique preventing postoperative hernia is a global controversial issue. Some studies have found that minimally invasive methods (LRP or RALP) display lower cumulative PIH rates compared to RRP [8, 9]. Nevertheless, a Nationwide, large population-based study demonstrated that no significant difference was found in risk of PIH between RRP and RALP with 11,212 patients [10]. Risk factors including age, body mass index (BMI), previous history of hernia or abdominal surgery, prostate volume, pelvic lymph node dissection (PLND), incontinence outcome, operative time, surgeon experience and patent processus vaginalis have been studied to be presumably associated with PIH previously [11–14], but no consensus was reached due to various inclusion criteria and population heterogeneity. Moreover, we assume that the surgical approaches should be associated with PIH incidence, but no researchers have studied PIH incidence taking both operative methods and approaches into consideration and no relevant data is available up to now. Thus, we performed a retrospective study to observe de novo hernia rate after prostatectomy and identified risk factors to guide the physicians taking prophylactic procedures for high-risk patients.

## Methods

### Patients selection and data collection

This study included all male patients who underwent RRP, LRP or RALP for PCa at The First Affiliated Hospital of Nanjing Medical University from June 2009 to September 2016. After receiving the ethical committees approval of our hospital with decision number 2019-SR-125, demographic and perioperative data including age, BMI, previous abdominal operations, diabetes mellitus history, hypertension history, prostate volume, previous hernia, operative methods, operative approach, preoperative Gleason, clinical T-stage, PLND situation, operative time, estimated blood loss, were extracted through retrospective database and medical records retrieval for statistical analysis. A total of 751 cases were eligible, composing of 59 RRP,638 LRP, and 54 RALP cases, the cumulative incidence of PIH rate was investigated accordingly. RRP was operated in extraperitoneal approach with a 10 cm midline incision below the umbilicus. LRP and RALP were operated in extraperitoneal or transperitoneal approach alternatively with 12 mm-Hg insufflation pressure and 5–6 trocar incisions. PLND was performed for those with high probability of lymph node invasion based on preoperative imaging and intraoperative conditions. Staging was defined according to the American Joint Committee on Cancer (AJCC) TNM staging system [15].

The primary endpoint is the occurrence of postoperative inguinal hernia. As all the chosen patients were followed up every 3 months in clinics with blood drawn and physical examination done routinely, those complaining of mass or discomfort in the groin area were transferred to hernia group of general surgery department for further diagnosis. The diagnosis of PIH was carried out based on clinical examinations and ultrasound imaging. No patients were lost to follow-up. Patients with PIH did not have any prior radiotherapy.

### Statistical analysis

Categorical variables and continuous variables were presented as proportion and mean ± standard deviation (SD) or median and interquartile range (IQR) respectively. Independent t-test was utilized for continuous variables that satisfy the normal distribution and homogeneity of variance (age, BMI, prostate volume) while Wilcoxon rank-sum test is used for those unsatisfied (operative time). Chi-square test/Fisher’s exact test was used for categorical variables (previous abdominal operations, diabetes mellitus history, hypertension history, previous hernia, operative methods, operative approach, preoperative Gleason, clinical T-stage, PLND situation, estimated blood loss). Univariate and multivariate cox hazard regressions analysis were used to identify risk factors predisposing to the occurrence of PIH. Kaplan–Meier analysis was used to evaluate hernia-free rate according to statistically significant variables obtained from COX hazard regression models. R version 3.4.1(2017-06-30) was used for all statistical analysis, *P* value < 0.05 was deemed to be statistically significant.

## Results

The median follow-up time of this study was 43 (range,2 to 104) months. Overall cumulative incidence of PIH was 7.1% (53/751), comprised of 15.3% (9/59), 6.7% (43/639), 1.9% (1/54) for RRP, LRP and RALP, respectively. The median time to develop a PIH was 12 (range 4 to 50) months. Among the total 53 PIH cases, indirect and direct hernia were observed in 47 (88.8%) and 6 (11.2%) patients, respectively. Moreover, 37(69.8%) hernias were in right side while 13 (24.5%), and 3 (5.7%) cases were found on the left and both sides, respectively.

Demographic and perioperative characteristics of all 751 patients involved in this study were shown in Table 1. There was no significant difference in BMI, previous abdominal operations, diabetes mellitus history, hypertension history, operative approach, PLND situation, operative time and estimated blood loss between the hernia group and hernia-free group. However, age was higher than those in hernia-free group (71.0 ± 5.6 vs 68.6 ± 6.7, *P* = 0.01). Gleason score (*P* = 0.049) and clinical T-stage (*P* = 0.038) in hernia-free group were more statistically advanced compared to the hernia group.

**Fig. 1:**
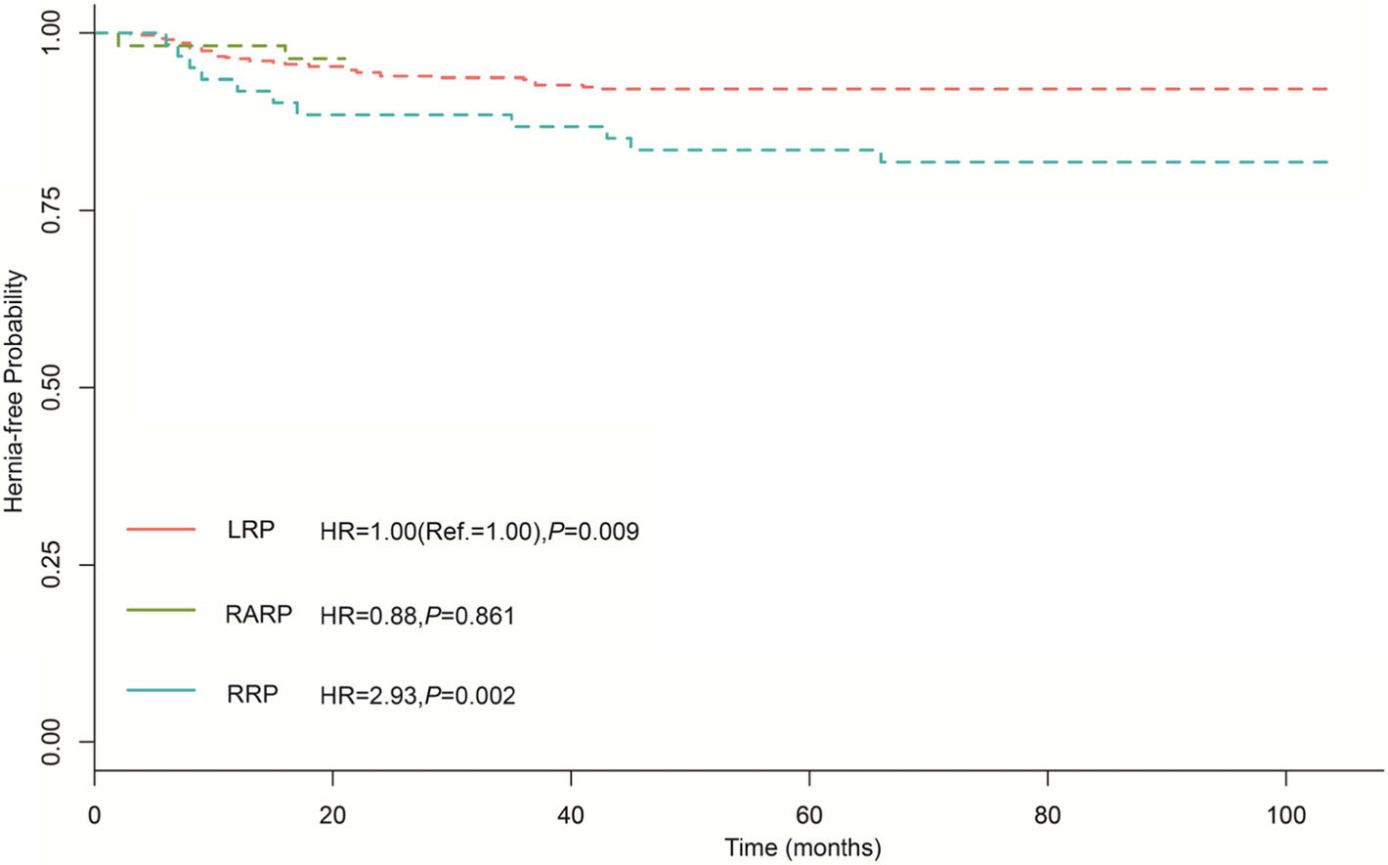
The Kaplan–Meier PIH-disease-free progression stratified by operative methods

**Table 1:** demographic and perioperative characteristics between hernia group and no-hernia group

Characteristic	hernia group (*N* = 53)	no-hernia group (*N* = 698)	total (*N* = 751)	statistic (*t、W、χ*^*2*^)	*P*
age-(years)					
mean ± sd	71.00 ± 5.59	68.57 ± 6.70	68.74 ± 6.65	*t* = −2.575	**0.01020**
range	54–81	28–86	28–86		
BMI-(kg/m^2^)					
mean ± sd	23.34 ± 0.51	23.97 ± 2.96	23.96 ± 2.93	*t* = 0.300	0.765
range	22.99–23.70	16.89–30.82	16.90–30.82		
previous abdominal operations -no.(%)					
yes	11 (20.8)	128 (18.4)	139 (18.6)	*χ*^*2*^ = 0.182	0.670
no	42 (79.2)	568 (81.6)	610 (81.4)		
diabetes mellitus history -no.(%)					
yes	11 (20.8)	88 (12.6)	99 (13.2)	*χ*^*2*^ = 2.825	0.093
no	42 (79.2)	608 (87.4)	650 (86.8)		
hypertension history -no.(%)^a^					
yes	25 (47.2)	274 (39.4)	299 (39.9)	*χ*^*2*^ = 1.250	0.264
no	28(52.8)	422 (60.6)	450 (60.1)		
previous hernia -no.(%)^a^					
yes	5 (9.4%)	31 (4.5)	36 (4.8)	*χ*^*2*^ = 2.659	0.103
no	48 (90.6)	664 (95.5)	712 (95.2)		
preoperative Gleason^a^					
< 7	17 (32.1)	131 (19.1)	148 (20.0)	*χ*^*2*^ = 6.048	**0.049**
7	25 (47.2)	340 (49.5)	365 (49.3)		
> 7	11 (20.8)	216 (31.4)	227 (30.7)		
T-stage-no.(%)^a^					
T1	11 (20.8)	69 (10.0)	80 (10.8)	*χ*^*2*^ = 10.174	**0.038**
T2	32 (60.4)	394 (57.1)	426 (57.3)		
T3	9 (17.0)	145 (21.0)	154 (20.7)		
T4	1 (1.9)	80 (11.6)	81 (10.9)		
PLND-no.(%)^a^					
yes	31 (58.5)	423 (61.3)	454 (61.1)	*χ*^*2*^ = 0.164	0.686
no	22 (41.5)	267 (38.7)	289 (38.9)		
operative methods -no.(%)					
LRP	43 (81.1)	594 (85.2)	638 (84.8)	*χ*^*2*^ = 8.442	**0.038**
RALP	1 (1.9)	53 (7.6)	54 (7.2)		
RRP	9 (17.0)	50 (7.2)	59 (7.9)		
operative approach -no.(%)^a^					
extraperitoneal	26 (49.1)	268 (38.5)	294 (39.1)	*χ*^*2*^ = 2.404	0.301
transperitoneal	27 (50.9)	429 (61.5)	456 (60.7)		
operative time-(min)					
median(P25, P75)	150.0 (120.0,215.0)	175.0 (130.0,210.0)	170 (130,210)	*W* = 16,154	0.151
estimated blood loss-(ml)^a^					
< 500.0	24 (66.7)	311 (66.2)	335 (66.2)	*χ*^*2*^ = 0.060	0.970
< 1000.0	10 (27.8)	128 (27.2)	138 (27.3)		
< 2000.0	2 (5.6)	31 (6.6)	33 (6.5)		

*SD* Standard deviation, *LRP* Laparoscopic radical prostatectomy, *RARP* Robot-assisted laparoscopic radical prostatectomy, *RRP* Retropubic radical prostatectomy, *PLND* Pelvic lymph node dissection

^a^ some data missing

**Fig. 2:**
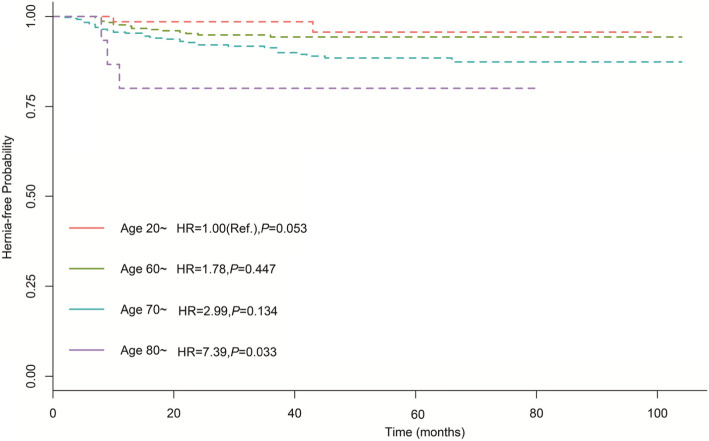
The Kaplan–Meier PIH-disease-free progression stratified by age

**Fig. 3:**
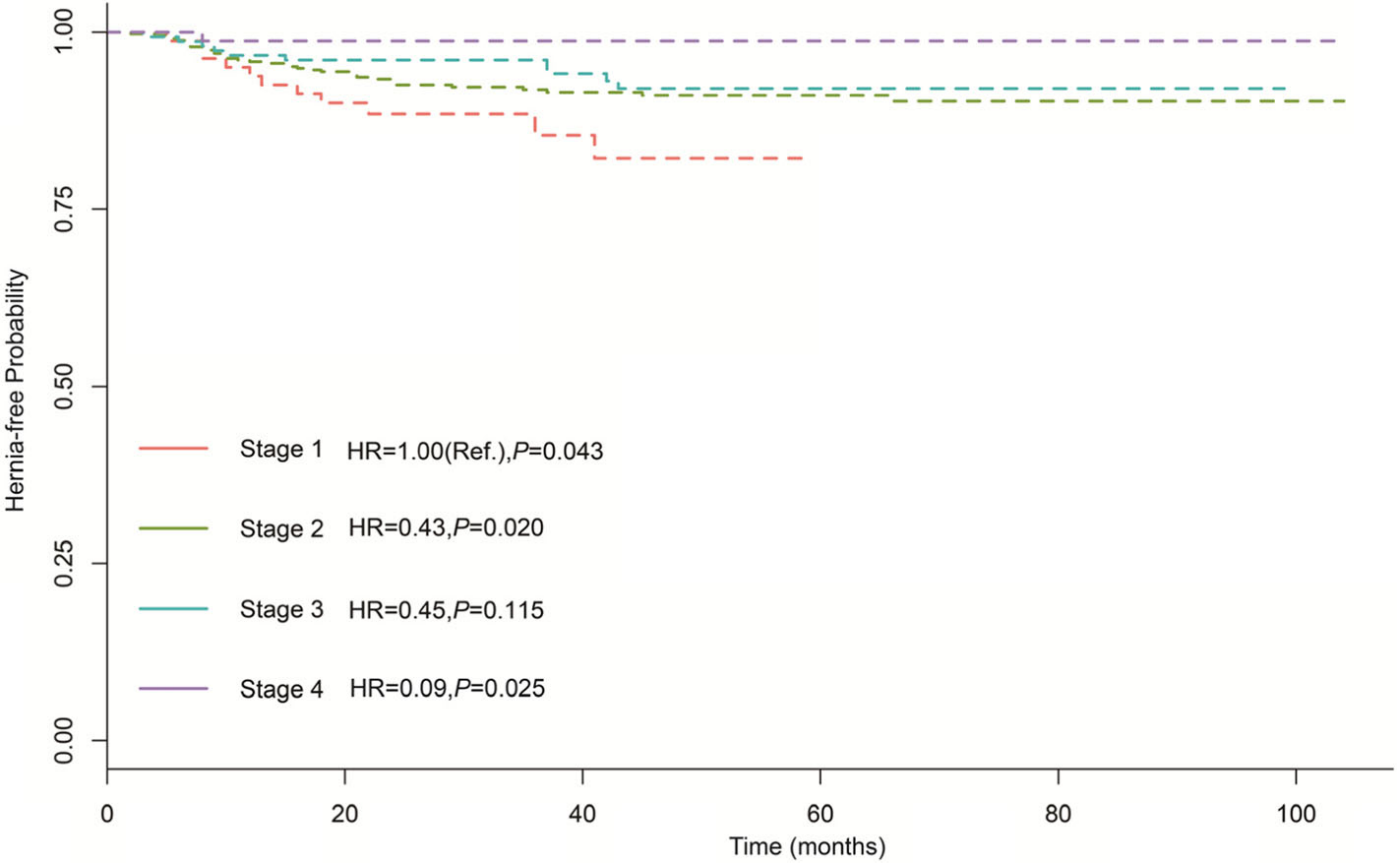
The Kaplan–Meier PIH-disease-free progression stratified by clinical T-stage

The results of the univariate and multivariate cox hazard regressions were presented in Table 2. On univariate analysis, age, diabetes mellitus history, operative methods, operative approach, and clinical T-stage were brought into multivariate analysis under criterion of *P* < 0.2. Finally, age and RRP approach were identified as independent risk factors for PIH (Adjusted hazard ratio 7.39, 95% CI: 1.18–46.39, and 2.93, 95% CI: 1.47–5.84). Additionally, clinical T-stage is a protective factor with adjusted hazard ratio 0.09 (95% CI 0.01–0.73). The Kaplan–Meier hernia-free rate regarding age, operative methods and clinical T-stage were presented in Figs. 1, 2 and 3, which showed PIH occurrence was significantly increased in RRP and senior age patients. With regards to clinical T-stage, T4 patients shared a lower PIH rate.

**Table. 2:** Univariate and multivariate Cox hazard regressions analysis of population and clinical factors for PIH

Characteristic	*Univariate*		*Multivariate*	
	HR(95%CI)	*P*值	*HR(95%CI)*	*P*值
age (years)				
20~	1.00(Ref.)	0.172	1.00(Ref.)	0.053
60~	1.32 (0.27–6.29)	0.729	1.78 (0.40–7.82)	0.447
70~	2.33 (0.53–10.22)	0.262	2.99 (0.71–12.58)	0.134
80~	5.88 (0.72–47.97)	0.098	7.39 (1.18–46.39)	**0.033**
previous abdominal operations				
no	1.00(Ref.)			
yes	0.95 (0.38–2.33)	0.902		
diabetes mellitus history				
no	1.00(Ref.)		1.00(Ref.)	
yes	0.54 (0.24–1.22)	0.140	0.65 (0.32 ~ 1.30)	0.224
hypertension history				
no	1.00(Ref.)			
yes	0.94 (0.47–1.89)	0.857		
previous hernia				
no	1.00(Ref.)			
yes	0.82 (0.24–2.87)	0.759		
preoperative Gleason				
< 7	1.00(Ref.)	0.638		
7	0.69 (0.31–1.53)	0.358		
> 7	0.70 (0.24–2.04)	0.509		
T-stage				
T1	1.00(Ref.)	0.291	1.00(Ref.)	**0.043**
T2	0.54 (0.22–1.34)	0.184	0.43 (0.21–0.87)	**0.020**
T3	0.41 (0.15–1.94)	0.339	0.45 (0.17–1.21)	**0.115**
T4	0.13 (0.01–1.25)	0.077	0.09 (0.01–0.73)	**0.025**
PLND				
no	1.00(Ref.)			
yes	1.58 (0.75–3.32)	0.23		
operative methods				
LRP	1.00(Ref.)7	0.110	1.00(Ref.)	**0.009**
RALP	0.77 (0.18–3.19)	0.480	0.88 (0.21–3.69)	**0.861**
RRP	2.28 (1.18–4.44)	0.053	2.93 (1.47–5.84)	**0.002**
operative approach				
extraperitoneal	1.00(Ref.)		1.00(Ref.)	
transperitoneal	0.50 (0.22–1.12)	0.093	0.74 (0.40 ~ 1.39)	0.35
estimated blood loss				
< 500.0	1.00(Ref.)	0.552		
< 1000.0	0.68 (0.29–1.59)	0.376		
< 2000.0	0.50 (0.10–2.43)	0.390

In general, hernia repair with mesh implantation was performed in 51 of 53 PIH patients; the remaining 2 cases were followed-up regularly due to mild symptoms. There was no case of emergency hernia surgery. No complication was seen after hernioplasty.

## Discussion

Our data showed that: (1) 7.1% (53/751) of patients developed PIH after RRP, LRP or RALP treatment and 83.0% (44/53) of them occurred in the first 2 years. (2) Right indirect inguinal hernia was the dominant type which developed in 34 of all 53 (64.2%) patients. (3) RRP and senior age were prone to promote the formation of PIH and advanced preoperative clinical T-stage, however, was the protective factors for PIH which needs further consolidation.

Hernia compromise the quality of life for the common morbidity such as pain, intestinal dysfunction, ischemia of the hernia contents and may even lead to the incarcerated or strangulated hernia which require surgery in emergency. Nearly 9% patients with inguinal hernia require urgent surgery [16]. Additionally, a bowel resection will be inevitable in 15% of incarcerated hernias [17].. Consequently, PIH should be deemed as a complication we could not neglect. Takashige Abe et al. [7] reported a PIH incidence with 17 and 14% after RRP and LRP compared with 1.4% in radiotherapy group. Lee et al. found an inguinal hernia rate of 3.4% after RALP in a 1026-cases review [18]. Our data is consistent with previous literatures. The present data demonstrated that the incidence of PIH in RRP group was much higher than in both LRP and RALP groups. The length of low midline incision was recognized as a risk factor for occurrence of PIH in 2008 [19]. We, therefore, attributed this results to the advantages of minimally invasive technique (LRP or RALP) for making five to six 8-12 mm trocar incisions instead of one 10-12 cm incision in RRP presumptively.

The precise mechanism of PIH occurrence remains obscure; many antecedent conjectures had been discussed. The dissection of retropubic (Retzius) space is ineluctable during prostatectomy in all technique, Nomura suggested that transversalis fascia, posterior layer of the rectus sheath and subjacent endopelvic fascia, in where the weakest part of posterior inguinal wall locate, are damaged during retropubic exposure. Additionally, stretching of Hesselbach’s ligament results in loosening the strength of internal ring and the formation of PIH [20]. Since the invention of laparoscopic and robotic assisted technique, it rapidly took place of RRP and become the most prevalent prostatectomy option, which provides surgeons with a wider vivid 3D view of operative area, magnified anatomy of pelvis, flexible arm allowing them to achieve exposure with less damage and dissection to the tissues and vessels. Compared to traditional RRP methods, maximum conservation of the physiological construction of the Retzius space and less alteration of myopectineal orifice action were gained owing to accurate controllability of minimally invasive instruments [21]. Eventually, we prefer LRP and RALP regarding its better oncological and complication outcomes [22].

The causative reason for PIH is multifactorial. So far, only one meta-analysis had been published, in which the increasing age, low body mass index, subclinical inguinal hernia, previous inguinal hernia repair and anastomotic stricture were recognized to be predisposed to higher incidence of PIH [23]. However, only the senior age was identified as a risk factor to PIH occurrence. Similar to the general population [24], the risk of PIH increases with age as well. Admittedly, aging and regression of the muscles and connective tissues around deep inguinal ring moderate supportive strain of abdominal wall. To our surprise, advanced clinical T-stage appeared to be the protective factor for PIH development. We suggested that it is probably related to older age in T1/T2/T3 patient, 69.04 ± 6.41 compared to 67.06 ± 6.94 in T4 patients(*P* = 0.009). We perceived that longer operative time would let everlasting insufflation pressure cause more damage to transversalis fascia, but no significant relation was observed.

Our data also showed a dominance of right-side hernia after prostatectomy, more than half (69.8%) of patients with PIH on right groin. The same result was found by other investigator likewise [3, 25]. It was hypothesized that the adherence of sigmoid colon and pelvic floor near to left internal ring protect celiac contents from herniating [26].

The best way to prevent PIH after prostatectomy remains debatable. Hori et al. bluntly detached peritoneum at the internal ring so as to isolate spermatic cord, merely 3% of RRP patients in prevented group developed PIH compared to 19% in non-prevented group [27]. Another effective procedure, opening the spermatic sheath and releasing approximately 5 cm bilateral vas deferens and spermatic vessels from the peritoneum, resulted in 0.87% PIH rate in prevented group while 15.7% non-prevented patients suffered from PIH, was introduced by Koike in 2013 [28]. The conceivable principle of these manipulations was that the scar tissues formed by isolating the spermatic cord could easily help to strengthen the internal ring. All these techniques should be viewed as valuable adjuncts for high-risk patients in need of prostatectomy.

Our study had several limitations that should be addressed. The number of patients per groups was not homogenously divided as this was a retrospective study with results based on database and medical records. Moreover, imaging examination was not applied for every prostatectomy patient, the asymptomatic PIH may not be detected, so the true incidence rate of PIH should be higher. Besides, as a potential risk factor, prostate volume information was incomplete to take further statistical analysis. Lastly, RALP technique was introduced to our centre for merely 2 years, case number was not as many as LRP group, long-term investigation will be necessary in the future.

## Conclusion

Post-prostatectomy inguinal hernia remains a worrisome complication. We discovered that patients undergoing RRP technique and/or those of senior age are at the highest risk of PIH. As a result, we advocate that those patients are approached with additional care and all available measures to reduce PIN should be employed.

## Data Availability

All data generated or analysed during this study are included in this published article.

## Abbrevations

PCa: Prostate cancer
PSA: Prostate-specific antigen
RRP: Retropubic radical prostatectomy
LRP: Laparoscopic prostatectomy
RALP: Robotic assisted laparoscopic prostatectomy
PIH: Postoperative inguinal hernia
BMI: Body mass index
PLND: Pelvic lymph node dissection
